# Immune cell subset differentiation and tissue inflammation

**DOI:** 10.1186/s13045-018-0637-x

**Published:** 2018-07-31

**Authors:** Pu Fang, Xinyuan Li, Jin Dai, Lauren Cole, Javier Andres Camacho, Yuling Zhang, Yong Ji, Jingfeng Wang, Xiao-Feng Yang, Hong Wang

**Affiliations:** 1 Center for Metabolic Disease Research, Lewis Kats School of Medicine, Temple University, Medical Education and Research Building, Room 1060, 3500 N. Broad Street, Philadelphia, PA 19140 USA; 2 Department of Pathology and Laboratory Medicine, University of Pennsylvania, Philadelphia, PA USA; 3 Department of Pharmacology, Lewis Kats School of Medicine, Temple University, Philadelphia, PA USA; 4 Cardiovascular Medicine Department, Sun Yat-Sen Memorial Hospital, Sun Yat-Sen University, Guangzhou, 510120 China; 5 Key Laboratory of Cardiovascular Disease and Molecular Intervention, Nanjing Medical University, Nanjing, China

**Keywords:** Immune cell subset differentiation, T cell, B cell, Dendritic cell, Monocyte, Macrophage, Cardiovascular disease, Cancer

## Abstract

Immune cells were traditionally considered as major pro-inflammatory contributors. Recent advances in molecular immunology prove that immune cell lineages are composed of different subsets capable of a vast array of specialized functions. These immune cell subsets share distinct duties in regulating innate and adaptive immune functions and contribute to both immune activation and immune suppression responses in peripheral tissue. Here, we summarized current understanding of the different subsets of major immune cells, including T cells, B cells, dendritic cells, monocytes, and macrophages. We highlighted molecular characterization, frequency, and tissue distribution of these immune cell subsets in human and mice. In addition, we described specific cytokine production, molecular signaling, biological functions, and tissue population changes of these immune cell subsets in both cardiovascular diseases and cancers. Finally, we presented a working model of the differentiation of inflammatory mononuclear cells, their interaction with endothelial cells, and their contribution to tissue inflammation. In summary, this review offers an updated and comprehensive guideline for immune cell development and subset differentiation, including subset characterization, signaling, modulation, and disease associations. We propose that immune cell subset differentiation and its complex interaction within the internal biological milieu compose a “pathophysiological network,” an interactive cross-talking complex, which plays a critical role in the development of inflammatory diseases and cancers.

## Background

The innate and adaptive immune system have been traditionally recognized mostly as the cellular response of immune cells, including T cells (TC), B cells (BC), dendritic cells (DC), monocytes (MC), and macrophages (MØ). During recent decades, molecular immunology research advanced this classical immunology concept with the discovery of subsets of each immune cell lineage. Between 1983 and 1992, the first major immune cell subsets were discovered [1–5]. Following these important breakthroughs, immune subset cell heterogeneity was proposed and its research has been continually flourishing. To date, more than 80 immune cell subsets are recognized [6]. Multiple cytokines and transcription factors have been discovered to control immune cell subset development. Moreover, the concept of immune cell subset plasticity was proposed. For example, pathological conditions could re-shape physiological regulatory T cells (Treg) into pathological Treg that have weakened immunosuppressive functions [7]. Here, we summarize our current knowledge regarding immune cell heterogeneity with an emphasis on their molecular characteristics and potential contributions to cardiovascular diseases (CVD) and cancers. We hypothesize that different immune cell subsets compose a “pathophysiological network” and an interactive cross-talking complex, which is crucial in the development of human diseases.

## General processes of hematopoietic stem cell differentiation

**Fig. 1:**
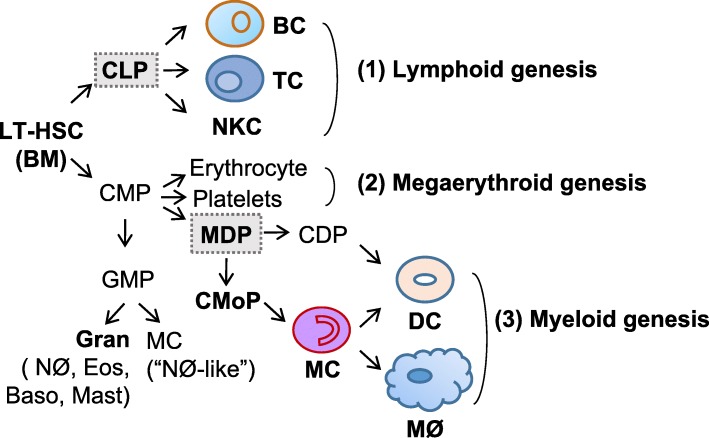
General processes of hematopoietic stem cell differentiation. Long-term hematopoietic stem cells (LT-HSC) differentiate into common lymphoid progenitors (CLP) and common myeloid progenitors (CMP). CLP are committed to lymphoid genesis and differentiate into B cells (BC), T cells (TC), and natural killer T cells (NKT) (1). CMP are committed to megaerythroid genesis and could differentiate into erythrocytes and platelets (2). CMP could also differentiate into granulocyte-macrophage progenitors (GMP), and then differentiate into granulocytes (neutrophils (NØ), eosinophils, basophils, and mast cells) and “NØ-like” MC. In addition, CMP could differentiate into monocyte-dendritic progenitors (MDP), which are committed to myeloid genesis and differentiate into common dendritic cell progenitor (CDP)-derived plasmocytoid dendritic cells (pDC) and common monocyte precursor (cMoP)-derived monocytes (MC), which further differentiate into monocyte-derived DC (mDC) and macrophages (MØ) (3)

The classical hematopoietic process has been updated in recent years, with the addition of committed common monocyte progenitors (CMoP) and granulocyte–macrophage progenitor (GMP)-independent monocyte-macrophage/dendritic lineage-restricted progenitor (MDP) arising from common myeloid progenitors (CMP) [8]. Nevertheless, it remains in agreement that the hematopoietic process is conserved among vertebrates [9] and that all hematopoietic lineages are derived from the long-term hematopoietic stem cell (LT-HSC) in bone marrow (BM) (Fig. 1). LT-HSC divisions can result in self-renewal or differentiation into multipotent and lineage-committed hematopoietic progenitor cells, such as common lymphoid progenitors (CLP), CMP, and GMP, all of which lack or have limited self-renewal capacity. CLP differentiate into lymphoid cells (T, B, and natural killer cells), while CMP give rise to megaerythroid cells (platelets and erythrocytes) when GATA-1 is highly expressed. CMP can differentiate into GMP with the expression of “myeloid factor” C/EBPα [10], or differentiate into MDP [8]. GMP could produce “neutrophil-like” inflammatory MC as well as granulocytes. By contrast, MDP could differentiate into common dendritic cell progenitor (CDP) and then DC. MDP also give rise to cMoP, which is a clonogenic, MC and MØ-restricted progenitor cell type [11]. cMoP further develop into MC, MC-derived MØ, and MC-derived DC. The balance among different immune cell subset differentiation is tightly controlled, the dysregulation of which contributes to both CVDs and cancers. However, significant future studies are still needed to identify more accurate markers for theses immune cell subsets and analyze their differentiation process systemically. Moreover, novel unidentified progenitor subsets still await discovery.

## Lymphoid genesis and subset differentiation

### TC development and subset differentiation

**Fig. 2:**
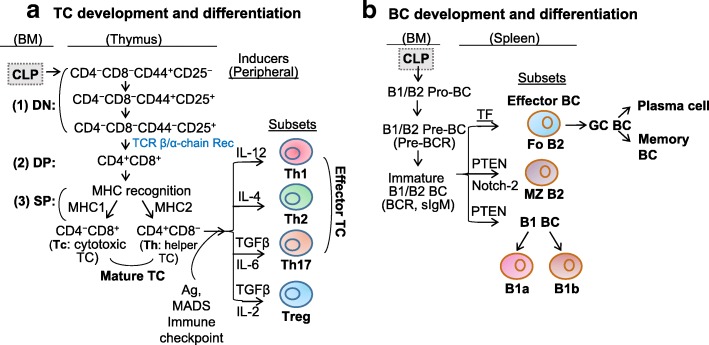
Lymphoid genesis and subset differentiation. **a** Differentiation of T cells (TC). Common lymphoid progenitors (CLP) migrate from bone marrow (BM) to thymus entering double-negative (DN, CD4-CD8-) stage (1). These cells initially express adhesion molecule CD44 and then α-chain of the interleukin (IL)-2 receptor (CD25), eventually lose CD44 and maintain CD25, rearrange T cell receptor (TCR) β chain, and then enter double-positive (DP, CD4+CD8+) stage (2), a transition stage of TC maturation. The DP TC then lose their membrane expression of CD25, rearrange their α chain, generate a complete αβ TCR, which has the capacity to recognize host major histocompatibility complex (MHC) molecules (positive selection), therefore survives and enters single positive (SP, CD4+, or CD8+) stage (3). After TC bind to MHCI, they become CD8+ TC and are termed as cytotoxic TC (Tc), whereas those binding to self-peptide–MHCII become CD4+ TC and are called as Naïve TC (T0). These SP TC then undergo “negative selection” to eliminate those that recognize MHC that bound to self-peptides, thereby completing the process of TC maturation. T0 cells can differentiate into effector TC (T helper cells Th1, Th2, Th17) and regulatory T cells (Treg) under the regulation of antigen (Ag) presentation, immune checkpoint, cytokine inducers, and metabolite-associated danger signal (MADS), and produce functional cytokines. **b** Differentiation of B cells (BC). In BM, B1/B2-specific CLP first differentiate into B1/B2 progenitor BC (pro-BC), B1/B2 precursor BC (pre-BC) with assembled pre-B cell receptor (BCR) and then became immature B1/B2 BC that express BCR and secrete IgM. Immature B1/B2 BC then migrate to spleen and differentiate into follicular (Fo) B2 BC, marginal zone (MZ) B2 BC, or mature B1 BCs, depending on the transcription factors that are induced by different signals. Fo BC can generate GC BC with follicular DC (a stromal cell) retained-Ag encounter and Tfh help. An affinity-matured Ab response then produce durable memory BC with high affinity to foreign Ag, as well as long lived plasma cells, which can secrete large quantities of Ab. B2 BC constitute the majority of splenic BCs. Mature B1 BC further differentiate into B1a secreting IgM and IL-10, and B1b producing IgM

CLP migrates from BM to the thymus and differentiate into TC (Fig. 2a). Upon entering the thymus, CLP first become double-negative (DN) thymocytes which do not express the TC co-receptors CD4 and CD8, or T cell receptor (TCR). During the DN stage, CD4^−^CD8^−^ thymocytes gradually lose expression of adhesion molecule CD44 and gain expression of α chain of the IL-2 receptor CD25 on their cell surface, which selects cells that have successfully rearranged their TCR-β chain locus. Then, the DN TC starts to express pre-TCR, which is composed of the re-arranged β chain and pre-α chain. Pre-TCR expression leads to the double-positive (DP) transition with the expression of both CD4 and CD8 (CD4^+^CD8^+^) and the subsequent replacement of pre-TCR with TCR (both α chain and β chain re-arranged) [12].

The fate of the DP TC depends on signaling that is mediated by interaction of TCR with self-peptide presented by major histocompatibility complex (MHC) classes. Too little signaling results in delayed apoptosis (death by neglect, positive selection), while too much signaling can promote acute apoptosis (negative selection) [13, 14]. These DP TC then interact with thymus cortical epithelial cells that express a high density of self-peptide-MHC complexes. DP TC that bind to self-peptide–MHCI complexes become cytotoxic CD8^+^ TC, which lose CD4 expression because of signal-interrupted CD8 downregulation. Whereas those that bind to self-peptide–MHCII ligands become naïve CD4^+^ TC which lose CD8 expression because of continued CD8 downregulation. These single-positive (SP) TC are now mature and migrate to peripheral lymphoid sites. Cytotoxic CD8^+^ TC destroy virus-infected cells and tumor cells, and they are also implicated in transplant rejection. Helper CD4^+^ TC assist other leukocytes in immunologic processes, while regulatory CD4^+^ TC maintain immunological tolerance.

The pro-inflammatory Th1 and anti-inflammatory Th2 were discovered in 1989 by Mosmann and Coffman [1]. Then, this Th1/Th2 paradigm was expanded after the discovery of the additional subsets of Th cells, including Treg [15] and IL-17-producing Th17 [16], in the mid-1990s and 2005, respectively.

The subset differentiation of naïve CD4^+^ TC is determined by antigen presented by the antigen presenting cell (APC), immune checkpoints co-signaling, cytokines, and metabolism-associated danger signal (MADS) [17]. Different cytokines direct TC to polarize into subtypes. Interleukin (IL)-12 was discovered in the early 1990s to play a major role for the generation of Th1 cells [18]. At the same time, IL-4 was discovered as a critical cytokine for the generation of Th2 cells in vitro [19]. In 2006, CD4^+^ TC was found to express IL-17 (designated Th17 cells, a third subset of T helper cells) in response to the combination of IL-6 and transforming growth factor beta (TGF-β) [20]. Treg are generated when naïve CD4^+^ TC are primed with IL-2 and TGF-β [21]. Treg express high levels of forkhead box P3 (FOXP3) and acquire the capacity to suppress TC response. Due to limited information on other CD4^+^ TC subsets, including Th9, follicular T (Tfh), and Th22 cells, we only include their characterization information (Table 1).

**Table 1:** Characterization of lymphoid cell subsets

Subsets		Markers	Frequency	Cytokines	Functions
TC	Th1	IL-2^+^TNF-β^+^IFN-Ɣ^+^	20% in blood CD4+ TC	IL-2, IL-12IFN-γ, TNF-α	↑MØ↑Cell-mediated immunity
	Th2	IL-4^+^IL-5^+^IL-10^+^IL-13^+^	2% in blood CD4+ TC	IL-4, IL-5, IL-10 IL-25, IL-13	↑Ab, Eos↓MØ function
	Th17	IL-17^+^RORγt^+^	0.5% in blood CD4+ TC	IL-21, IL-22, IL-24IL-26, IL-17A, IL-17F	Defend host↑Autoimmune disease
	Treg	Foxp3^+^IL-10^+^	5% in PBMC	IL-10, TGF-β	↓Autoimmune disease
	Tfh	CXCR5^+^	13.5%in blood CD4+ TC	IL-21	↑BC activation and functional differentiation
	Th22	AHR^+^CCR4^+^CCR6^+^CCR10^+^	0.05%In blood CD4+ TC	IL-17, IL-22	↓Immune activation
BC	Fo B2	CD21/35^int^CD23^+^CD24^low^CD62L^+^CD93-IgM^low^IgD^high^	4.3% in blood CD19+ BC	IgD, IgM	↑Adaptive response
	MZ B2/B1-like	CD21/35^high^CD23-CD24^+^CD93^−^IgM^high^IgD^low^	17% in blood BC	IgM	Respond toblood-borne pathogen

CD4+ helper T cells (TC) can be subdivided into seven groups, which include T helper cells Th1, Th2, Th17, regulatory T cells (Treg), Th9, T follicular helper cells (Tfh), and Th22. Th1 drive autoimmune diseases, while Th2 synthesize interleukin (IL)-4, IL-5, IL-6, and IL-1, and facilitate antibody production. Th17 produce IL-17 and play critical roles in autoimmunity and inflammatory diseases. Treg are in charge of suppressing potentially deleterious activities of Th cells. Th9 protect hosts against helminthic infection and also mediate allergic disease. Tfh are known to regulate BC activation and functional differentiation. Th22-secrected IL-22 maintains intestinal epithelial barrier integrity and stimulates the secretion of antimicrobial peptides that limit bacterial dissemination and intestinal inflammation. Bone marrow (BM)-derived B cells (BC) develop into either follicular (Fo) BC or marginal zone (MZ) BC in the spleen. Fo BC participate in TC-dependent immune responses to protein antigens. MZ BC express high levels of CD21 and CD1d, and respond vigorously to blood borne pathogens. Both B-1a and B-1b BC seed the peritoneal and pleural cavities. While B-1a BC contribute to innate-like immune responses, B-1b BC contribute to adaptive immunity

Interestingly, the differentiation of CD4^+^ TC into lineages with distinct effector functions is not an irreversible event. Among all these CD4 subsets, Th2, Treg, and Th17 cells are more plastic than Th1 and they may not be stable [7]. For example, Th2 can convert to Th9 in response to TGF-β stimulation. Treg can switch to Th1 by T-bet, Th2 by interferon regulatory factor 4 (IRF4), Th17 by IL-6+IL21/STAT3, or Tfh by B cell CLL/lymphoma 6 (BCL-6). Similarly, Th17 can convert into interferon gamma (IFN-γ)-producing Th1 cells or IL-4-producing Th2 cells when stimulated with IL-12 or IL-4, respectively.

### BC development and subset differentiation

The stages of BC developmental pathway have been extensively characterized over the past years, revealing important growth factors and regulatory interactions. It was initially described by Lee Herzenberg in 1983 that murine B1 cells are a unique CD5^+^ BC subpopulation [2] distinguished from conventional B2 cells by their phenotype, anatomic localization, self-renewing capacity, and production of natural antibodies. As our understanding progresses, interesting differences between B1 and B2 cell subsets have become apparent (Fig. 2b). B1 and B2 development occurs in BM and spleen [22]. In BM, CLP develop through B1- or B2-specific pro-BC, pre-BC, and immature BC. During this differentiation process, rearrangements at the immunoglobulin locus result in the generation and surface expression of the pre-B cell receptor (pre-BCR), and finally a mature BCR (comprised of rearranged heavy- and light-chain genes) that is capable of binding antigen. During this stage, BC undergo a selection process to prevent any self-reactive cells. Cells successfully completing this checkpoint leave bone marrow, eventually maturing into predominant follicular B2 (Fo BC), marginal-zone B2 (MZ BC), B1a, and B1b BC. Fo BC can further develop into germinal center (GC) BC with follicular DC-retained antigen encounter and Tfh help [23]. An affinity-matured antibody response will produce durable memory BC with high affinity to foreign antigen, as well as long-lived plasma cells, which can secrete large quantities of antibody.

### NKC development and subset differentiation

Both human [24] and mouse [25] studies have shown that NKC can be differentiated from CLP. Three major human NK cell subsets can be distinguished in peripheral blood based on the expression levels of low-affinity Fc-receptor γ IIIA (CD16) and neural cell adhesion molecule (NCAM, CD56), namely, CD56^high^CD16^high/low^, CD56^low^CD16^low^, and CD56^low^CD16^high^. These subsets are more likely to be NKC at a different stage of maturation. CD56^high^CD16^high/low^ can be firstly differentiated from BM-derived CLP [26]. These CD56^high^CD16^high/low^ NKC stay within the lymph node and interact with DC or further mature into CD56^low^CD16^low^, and then became cytotoxic CD56^low^CD16^high^ NKC [27].

## Myeloid genesis and subset differentiation

### DC development and subset differentiation

**Fig. 3:**
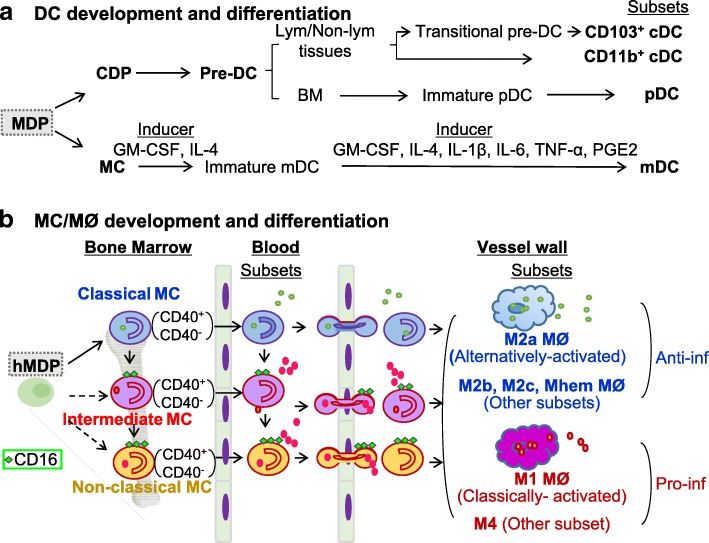
Myeloid genesis and subset differentiation. **a** Differentiation of dendritic cells (DC). Monocyte-dendritic progenitors (MDP) are the direct precursors to common dendritic cell progenitors (CDP) and monocytes (MC), which both give rise to DC lineages. In bone marrow (BM), CDP become precursor DC (pre-DC) and differentiate into immature plasmocytoid DC (pDC) and mature pDCs, and then exit the BM traveling through the blood to secondary lymphoid organs and non-hematopoietic tissues. In the lymphoid or non-lymphoid tissues, pre-DC become CD103+ classical/conventional DC (cDC) through transitional pre-cDC stage and CD11b+ cDC directly. In cultured system, MC differentiate into immature monocyte-derived DC (mDC) in the presence of interleukin (IL)-4 and granulocyte macrophage colony-stimulating factor (GM-CSF). Terminal differentiated mDC are induced upon stimulation with inflammatory cytokines (IL-1, IL-6, and tumor necrosis factor (TNF)) and prostaglandin E2 (PGE2). **b** Differentiation of monocytes (MC)/macrophages (MØ) in human. In the steady state, human classical MC can differentiate into intermediate MC, then patrolling non-classical MC. Classical MC have a high antimicrobial capability due to their potent capacity of phagocytosis. Intermediate MC secrete inflammatory cytokines and have inflammatory properties, whereas non-classical MC mainly patrol along endothelium. During inflammation, all the MC subsets are tethered and penetrate vessel wall and then mature into anti-inflammatory MØ (M2a, M2b, M2c, and Mhem) and inflammatory MØ (M1 and M4) in tissue. Classical, intermediate and non-classical MC can be further divided into CD40+ and CD40- MC subsets. CD40+ classical/intermediate MC are induced in cardiovascular disease (CVD) and further elevated with the progress of chronic kidney disease (CKD). Dashed arrows indicate potential differentiation pathways, while solid arrows indicate experimentally verified differentiation pathways

In 1973, Ralph Steinman and Zanvil Cohn [28, 29] discovered dendritic cells (DC). DC diversity was first acknowledged in 1992 [3]. Murine lymphoid organ DC consist of two subsets, defined by the presence or absence of CD8 expression, each with distinct immune functions [30]. After around another 20 years, CD103^+^ DC in nonlymphoid tissues was found to be lymphoid CD8^+^ DC equivalents, sharing several phenotypic features [31]. More recently, plasmacytoid DC (pDC) were uncovered. pDC morphologically resemble plasma cells but, upon exposure to viral stimuli, produce enormous amounts of IFN-α [32]. Importantly, pDC also differentiate upon stimulation into immunogenic DC that can prime TC against viral antigens. To distinguish pDC from the DC that are characterized by Steinman, the latter were renamed classical DC (cDC) and remain so today. Two main subsets of classical cDC along with pDC and MC-derived dendritic cell (mDC) all originate from MDP in BM (Fig. 3a). MDP firstly give rise to CDP and MC in BM. CDP can differentiate into pre-DC, and then become pDC and cDC. Pre-DC migrate from BM, via blood, to lymphoid and nonlymphoid tissues, and constantly replenish CD103^+^ and CD11b^+^ cDC [33]. In BM, pre-DC give rise to immature-pDC, and then pDC [34]. The basic helix-loop-helix (bHLH) transcription factor E2-2 is essential for pDC development in both mice and humans [35]. mDC can be generated by culturing human or mouse MC with granulocyte-macrophage colony-stimulating factor (GM-CSF) and other cytokines (IL-4, IL-1β, IL-6, tumor necrosis factor α [TNF-α], PGE2), which express high levels of CD11c and MHCII, and act as potent antigen-presenting cells [36].

### MC/MØ development and subset differentiation

Human MC are classified by the levels of their surface expression of CD14, CD16, and CD40 (CD14^++^CD16^−^, CD14^++^CD16^+^, CD14^+^CD16^++^, CD14^+^CD40^+^), which differ in their function and phenotype [37]. CD14^++^CD16^−^ MC, also called the classical MC, are the most prevalent MC subset in human blood. The CD14^++^CD16^+^ MC are intermediate MC that contribute significantly to atherosclerosis. The CD14^+^CD16^++^ MC are referred to as non-classical MC, which perform a patrolling function. In the steady state, classical MC can differentiate into intermediate MC, and then further differentiate into patrolling non-classical MC in circulation [38]. During inflammation, all MC subsets invade tissue and then mature to various MØ subsets according to environmental stimuli. Classical, intermediate, and non-classical MC can be further divided into CD40^+^ and CD40^−^ MC subsets. CD40^+^ classical/intermediate MC are induced in CVD and further elevated with the progress of chronic kidney disease (CKD) [39] (Fig. 3b).

Although in 1989, it was thought that human peripheral blood MC are a heterogeneous population of leukocytes, distinguishable by the expression of lipopolysaccharide (LPS) receptor CD14 and Fc-receptor γ IIIA CD16 [4], it was not confirmed in the murine circulation until 2001 [40]. Mouse MC subsets were originally defined by surface expression of CD11b, lymphocyte antigen 6 complex locus C1 (Ly6C), and CD62L and lacking CD11c and MHCII [41]. A second MC subset was discovered based on the higher expression of chemokine receptor CX3CR1 in *Cx3cr1*^GFP/+^ mice [42]. The expression of CX3CR1 was found to be opposite with granulocytic marker Gr1. Later, CX3CR1^low^ and CX3CR1^high^ MC (also termed as Gr-1^high^ and Gr-1^low^ MC) were defined as Ly6C^high^ and Ly6C^low^ MC, respectively. Ly6C is a glycosylphosphatidylinositol-anchored glycoprotein associated with homing to lymph nodes function. Gr-1 is composed of Ly6C and Ly6G subunits and its antibody recognize both Ly6C and Ly6G molecules. Ly6C is expressed on both MC and granulocytes, while Ly6G is exclusively expressed on granulocytes. Therefore, a better-justified approach to define mouse MC subsets is to use Ly6C^+^Ly6G^−^. This is also the reason that Ly6C, but not Gr-1, is used as mouse MC subset marker [43]. Currently, Ly6C^high^ MC is determined to be an inflammatory MC subset, corresponding to human conventional MCs. This subset can also be characterized with a set of surface markers and defined as CX3CR1^low^CCR2^+^CD62L^+^CCR5^−^. In contrast, the Ly6C^low^ MC is determined as a patrolling MC subset, corresponding to human non-classical MC and can be characterized as CX3CR1^high^, CCR2^−^, CD62L^−^, and CCR5^+^ [44]. The Ly6C^high^ and Ly6C^middle^ subsets perform pro-inflammatory functions and are considered the counterpart of human classical MC. The Ly6C^low^ subsets patrol along the vascular endothelium. They are involved in tissue repair, which resemble human non-classical MC. In steady state, Ly6C^+^ MC differentiate into Ly6C^−^ MC in the circulation. During inflammation, Ly6C^+^ MC are preferentially recruited into inflamed tissue and mature to different MØ subsets, based on various stimuli.

In 1973, Van Furth reported that pro-inflammatory stimuli elicit MC recruitment and MØ differentiation in tissue [45]. In1992, a new MØ subset, termed “alternative activation” of MØ, was identified from IL-4 induced differential gene expression [5]. By contrast, IFN-γ and LPS induced “classical activation” of MØ [46]. As shown in Fig. 3b myeloid precursor MDP give rise to MC within BM. Afterwards, MC exit to the blood, migrate to the tissues under inflammatory conditions, and further differentiate into MØ. Although BM-originated MC could be the precursors of MØ lineage, tissue MØ could also be derived from embryonic tissue [47], or converted from somatic cells, such as smooth muscle cells [48]. However, different developmental origins of MØ and their functional specificity remain largely unknown.

### Granulocyte development and subset differentiation

Mature granulocytes include neutrophils, eosinophils, basophils, and mast cells, which all develop from GMP. The maturation of neutrophil, eosinophil, and basophil proceeds from GMP to myeloblasts. When primary granules are visible, myeloblasts develop into neutrophil, eosinophil, and basophil myelocytes. When cell division ceases and secondary or specific granules begin to appear, they finally become mature neutrophils, eosinophils, and basophils [49]. Originally described in 1879 by Paul Ehrlich, the origin of mast cell is still a matter of debate [50]. Recently, researchers identified a mast cell progenitor that is derived from BM GMP [51]. The mast cell progenitors transiently circulate in the bloodstream and are capable of migrating to the peripheral tissues, where they mature into connective tissue mast cells or mucosal mast cells, following instructive signals from the tissue environment.

### Characterization of immune cell subsets

Most of the human immune cell subsets have mouse counterparts that share similar functions. We summarized the molecular and functional characterization of currently recognized immune cell subset.

### Characterization of lymphoid cell subsets (Table 1)

#### TC subset

The CD4^+^ TC subsets (Th1, Th2, Th17, Treg, Th9, Th22, and Tfh) differ in their extra-cellular and intracellular markers. Each TC subset releases specific cytokines that can have either pro- or anti-inflammatory functions, and they play either pathogenic or protective functions in human diseases. Th1 produce IFN-γ and TNF, and they can activate MØ, enhance cell-mediated cytotoxicity, mediate delayed-type hypersensitivity responses, and effectively respond to intracellular pathogens; Th2 release IL-4 (an important survival factor for BC and required for IgG1 and IgE production), IL-5, and IL-13; Th17 produce IL-17 (a strong inducer of autoimmune conditions, such as experimental encephalomyelitis (EAE)); Treg are characterized by the expression of FOXP3 (maintaining expression of FOXP3 transcription factor is needed for the suppressive function of Treg), and they secrete immunosuppressive cytokines IL-10 and TGF-β; Tfh are CD4^+^CXCR5^+^ TC [52, 53], and they are known to regulate the activation and differentiation of BC. They preferentially select BC that present high levels of peptide-MHCII and promote their extensive proliferation or differentiation to antibody-forming cells [54]; Th22 were recently distinguished from Th1 and Th17 by its unique IFNγ- and IL-17-independent IL-22 secretion [55]. It is characterized by the expression of CCR4, CCR6, CCR10, and aryl hydrocarbon receptor (AHR). Similar to Th17, Th22 have the capacity to acquire functional features of Th1 [56]. Moreover, within Th22, three more subsets were delineated in human liver, indicating the complexity of the immune cell subset system [57]; Th9 are defined by their production of IL-9 [58], although IL-9 is not unique to Th9. IL-9 is associated with host defense against intestinal nematode infection and the development of allergic responses. They could mediate protection against parasitic infections, while they could also induce allergic inflammation, asthma, and other autoimmune disease.

#### BC subset

Fo BC are activated in the spleen follicular niche by TC-dependent antigens of microbial origin. They receive synergistic signals via BCR, CD40, and toll-like receptors (TLRs) [59]. Activated Fo BC can further differentiate into GC BC, which express MHC-II and CD27 [60]. GC BC selection can lead to differentiation of antibody secreting plasma cells and memory BC [61]. Marginal zone B2 (MZ BC) are innate-like cells and appear to mediate TC-independent responses to antigens from blood-borne pathogens. MZ BC can also mediate the transport of antigen from immune complexes into splenic follicles, which is involved in TC-dependent BC responses. Moreover, MZ BC may participate in the immune responses to lipid antigen. Furthermore, they can shuttle between splenic follicle and marginal sinus, and the high level of CD21 expressed on MZ BC is presumably evolved to facilitate immune complex capture [59]. B1 BC population has not been clearly defined in humans [62], so our knowledge about the function of B1 BC is based on studies in rodents. B1 BC can contribute to the generation of IgM responses to TC-independent antigens such as phosphorylcholine, an antigen on many pathogenic bacteria [59]. While B-1a BC contribute to innate-like immune responses, B-1b BC contribute to adaptive immunity [63].

### Characterization of myeloid cell subsets (Table 2)

#### DC subset

DC express both MHCI and MHCII molecules [64], and they are unrivaled stimulators of TC. They process protein antigen and initiate antigen-specific cellular immune responses. Recent work has established that tissue DC consist of developmentally and functionally distinct subsets, including mDC, pDC, and cDC. As genetically distinct DC subsets, they arise from different bone marrow precursors. mDC are rapidly differentiated from MC and infiltrate into tissues in response to inflammation. Their role is therefore likely to be more specialized in expediting immune responses [65]. Recently, mDC are developed as immunotherapy target for treating cancer [66]. pDC accumulate mainly in blood and lymphoid tissues and they enter lymph node through blood circulation. Upon recognition of foreign nucleic acids, they produce massive amounts of type I IFN and acquire the capacity to present foreign antigens [67]. However, chronic inflammatory settings will exhaust pDC through IFN-I and TLR7 [68]. Human and murine cDC consist of two subsets as defined by the presence or absence of CD103, Xcr1, CD11b, and CD11c expression (CD11c^+^Xcr1^+^CD103^+^ or CD11c^+^CD11b^+^) [30]. CD11c is also expressed on MC and MØ, which complicates cDC characterization. CD11c^+^Xcr1^+^CD103^+^ cDC have superior ability over other cDC to present microbial antigens [69] and cell-associated antigens [69, 70] to CD8^+^ TC. CD11c^+^CD11b^+^ cDC are thought to have a predominant role in MHCII presentation to CD4^+^ TC [71].

**Table 2:** Characterization of myeloid cell subsets

Subsets		Markers	Frequency	Cytokines	Functions
DC	cDC	CD11c^+^MHCII^+^CD141^+^(BDCA3^+^)XCR1^+^CLEC9A^+^FLT3^+^CD103^+^	5~10% in blood	IL-12, IFN-III	↑IFN-IIICross-present Ag
		CD11c^+^MHCII^+^(BDCA1^+^)CD172a^+^CD11b^+^FLT3^+^	45–50% in blood	IL-23?	Present Ag
	pDC	BDCA2^+^LILRA4^+^CD45RA^+^	45–50% in blood	IFNα	Sense pathogenActivate IC
	mDC	MHCII^+^CD11c^+^CD86^+^CD40^+^CD80^+^CD83^+^CCR7^+^CD14^−^	Induced by inflammation	TNFα, iNOSIL-12, IL-23	Cross-present Ag
MC	Classical	CD14^+^CD16^−^CXCR1^+^ CXCR2^+^CD62L^+^	80–95% in MNC	ROS, NO, MPOIFN-I, IL-1α, TNFIL-6, IL-8, CCL2	Phagocytosis↑Inflammation
	Intermediate	CD14^+^CD16^+C^D64^int^CCR1^int^CCR2^int^ CX3CR1^int^ CD11b^int^ CD33^int^ CD115^int^ CD40^+^ CD54^+^ HLA-DR^+^	2–11% in MNC	ROS, NO, MPOIFN-I, IL-1α, TNFIL-6, IL-8 CCL2	↑Inflammation
	Non-classical	CD14^−^CD16^+^	2–8% in MNC	TNF, IL-1β, CCL3	Patrol, repair tissue
	CD40	CD14^+^CD40^+^	64% in PBMC	TNFα, IL-6	↑Inflammation
MØ	M1	iNOS^+^CXCL11^+^IL-12high IL-23^high^IL-10^low^	1% in gastric tissue	IL-6, TNFαiNOS, IL-12	Microbicidal↑Inflammation
	M2a	FIZZ1^+^Arg1^+^IL-12^low^IL-23^low^	1% in gastric tissue	IL-10	↓InflammationHeal woundRepair tissue
	M2b	CD80^high^CD14^high^HLA-DR^low^IL-12^low^IL-23^low^	0 in PBMC	IL-10	Activate Th2↓Inflammation
	M2c	CD86^low^HLA-DR^low^CD163^+^TLR4^high^IL-12^low^IL-23^low^	2.4% in CD68^+^ MØ	CCL18	↓InflammationDeposit matrixRemodel tissue
	M4	MMP7^+^MR^+^S100A8^+^CD68^+^	31.7% in CD68^+^ MØ from coronary artery	CD86, IL-6, TNFα	↑Inflammation
	Mhem	HOMX1^+^CD163^+^	25% in thrombosis	IL-10	↓Lipid accumulationRetain iron, ↓Inflammation

Four kinds of dendritic cells (DC) can be defined in human based in part on their functional specialization: monocyte-derived DC (mDC), CD103+ classical/conventional dendritic cell (cDC), CD11b + cDC, and plasmocytoid dendritic cell (pDC). mDC exhibit a strong costimulatory capacity for TC activation. cDC are the most efficient cell type for priming and functional polarization of TC. pDCs can secrete high concentrations of interferon (IFN)-I (mainly IFN-α). In humans, there are three populations of monocytes (MC), as defined by the expression of CD14 and CD16 (CD14++CD16−, CD14+CD16+, and CD14+CD16++). The CD14++CD16− MC represent 80% to 90% of blood MC, express high levels of the chemokine receptor C-C chemokine receptor type 2 (CCR2) and low levels of CX3C chemokine receptor 1 (CX3CR1), and produce IL-10 rather than TNF and IL-1 in response to lipopolysaccharide (LPS) in vitro. CD14+CD16+ MC express the Fc receptors CD64 and CD32, have phagocytic activity, and are entirely responsible for the production of tumor necrosis factor-α (TNF-α) and IL-1 in response to LPS. In contrast, CD14+CD16++ MC lack the expression of other Fc receptors, are poorly phagocytic, and do not produce TNF-α or IL-1 in response to LPS. Our lab recently identified CD40+ MC as a stronger inflammatory subset related to chronic kidney disease (CKD). Macrophages (MØ) also display phenotypic heterogeneity. Depending on the stimuli, M0 MØ could polarize towards the pro-inflammatory M1 subset by lipopolysaccharide or IFN-γ, or towards the alternative M2a type by IL-4. M2b MØs are induced upon combined exposure to immune complexes and Toll-like receptor (TLR) ligands or IL-1 receptor agonists. M2c MØs are induced by IL-10 and glucocorticoids. Atheroprotective Mhem subset could be induced by hemoglobin, and highly express haem oxygenase 1 and CD163. Chemokine (C-X-C motif) ligand 4 drives differentiation of human specific M4 MØ, with unique expression of surface markers such as S100A8, mannose receptor CD206, and matrix metalloproteinase 7

#### MC subset

Contemporary studies have demonstrated that four subsets of MC reside in the peripheral circulation. These subsets are distinct in their functions and fates [72].

Human MC subpopulations include CD14^++^CD16^−^ (classical, which account for 80–90% of peripheral blood MC), CD14^++^CD16^+^ (intermediate), CD14^+^CD16^++^ (non-classical) subpopulations [73], and CD14^+^CD40^+^ (CD40) MC [39]. Classical MC are phagocytic with no inflammatory attributes. The intermediate subtype constitutes a very small percentage in circulation (under physiological conditions); they appear to be transitional MC that display both phagocytic and inflammatory function. The non-classical subtype displays inflammatory characteristics upon activation and presents antigen [74]. Our lab recently identified CD40^+^ MC as a stronger inflammatory MC subset and a better marker for CKD severity stage compared with intermediate MC subset [39]. We provided evidence suggesting that metabolic risk factors, such as hyperhomocysteinemia (HHcy), induced CD40 in MC, in a reverse (towards APC) co-stimulation manner [17].

The mouse Ly6C^middle+high^ inflammatory subset corresponds to human CD14^++^CD16^−^ classical MC, while the mouse Ly6C^low^ patrolling subset corresponds to human CD14^+^CD16^++^ non-classical MC [75]. Microarray-based gene expression profiling has confirmed that differential gene expression profiles observed in mouse monocyte subsets are conserved in human monocyte subpopulations [73, 76]. Mouse Ly6C^middle+high^ inflammatory MC circulate in the blood and egress into tissues following pathological insult, at which point they differentiate into MØ and DC, producing inflammatory cytokines and reactive oxygen species, stimulating effector TC proliferation, and mediating tissue repair [75]. Ly6C^low^ MC are characterized by long-range crawling along the luminal surface of small vessels, which allows them to survey for dying and infected cells and mediate their disposal [75]. They also play an important role in mediating live vaccine’s protective effects by dictating Tfh differentiation, germinal center formation, and protective antibody production via IL-1β [77]. Furthermore, both Ly6C^+^ and Ly6C^−^ MC can have two sources including GMP and MDP, with distinct gene expression signatures and functions [8].

#### MØ subset

MØ are highly heterogeneous cells that can rapidly change their function in response to local microenvironmental signals [78]. *Classically activated MØ* (M1 MØ) are activated by cytokines such as IFN-γ [79]. M1 MØ protect the host from a variety of bacteria, protozoa, and viruses, and they also play critical a role in antitumor immunity [78]. *Alternatively activated MØ* (M2a MØ) have anti-inflammatory function and regulate wound healing [78]. M2a MØ can secrete large amounts of IL-10 in response to Fc receptor-γ (FcγR) ligation [80]. Interestingly, M2a MØ resembles tumor-associated macrophages (TAM) in cancer, which promote tumor progression by stimulating tumor proliferation, invasion, and metastasis, and inhibiting TC-mediated antitumor immune response [81]. The *third subset M2b* are activated when their FcγRs bind to LPS [82, 83]. M2b MØ turn off their production of IL-12 and secrete IL-10. In addition, M2b MØ upregulate antigen presentation and, importantly, promote Th2 responses. The *fourth subset M2c* is induced by IL-10/TGF-β, which exhibit anti-inflammatory functions in vitro and protect against renal injury in vivo due to their ability to induce Treg [84]. The activation of the *fifth subset M4* MØ is M-CSF/CXCL4-dependent [85]. M4 MØ are weakly phagocytic and unable to efficiently phagocytize acetylated LDL (acLDL) or oxidized LDL (oxLDL) [86]. In the context of atherosclerosis, atherosclerotic lesions have been demonstrated to contain M4 MØ, suggesting that M4 MØ may play important roles in the pathology of atherosclerosis [85]. The *sixth subset Mox* is polarized upon oxidized phospholipid (ox-PL) 1-palmitoyl-2arachidonoyl-sn-glycero-3-phosphorylcholine treatment, which upregulate the expression of oxygenase-1 (HO-1) and thioredoxin reductase 1 (Txnrd1) [87]. This unique Mox MØ comprised 23% of the aortic CD11b^+^F4/80^+^ population from 30-week western diet-fed low-density lipoprotein receptor-deficient (*Ldlr*^−/−^) mice [87]. As Mox MØ express anti-oxidant enzymes HO-1 and Txnrd1, they exert anti-inflammatory actions on vasculature in vivo [85]. This proposed phenotype of Mox MØ closely resembles the phenotypes of the *seventh subset Mhem* MØ, which generated from hapto-hemoglobin complexes or oxidized red blood cells treatment [88]. CD163 and IL-10 are upregulated in an Nrf2-dependent manner in Mhem MØ [88]. Mhem MØ promote atherosclerosis development due its angiogenic, vessel permeability causing, and leukocyte attracting properties, through hemoglobin:haptoglobin/CD163/HIF1α-mediated VEGF induction [89].

## Representative immune cell subset changes in diseases (Table 3)

Immune cell subsets play a significant role in the development of various inflammatory diseases and cancers and we have summarized their changes during various disease development (Table 3). Atherosclerosis is a chronic inflammatory pathogenic process of arteries, which leads to cardiovascular diseases (CVDs), including myocardial infarction and stroke [90]. Here, we focused on the discussion of the immune cell changes in atherosclerosis-related diseases. In human studies, the percentage of TC was found to be higher in blood samples from carotid atherosclerotic patients than those from healthy subjects [91, 92]. Similarly, a higher number of subintimal TCs was observed in those patients with carotid atherosclerotic plaque (CAP) [92]. This is also the case in rheumatoid arthritis (RA) [93]. Consistently, the number of natural killer T cell (NKT) was increased in the blood from patients that underwent endarterectomy [91] and those that had colorectal cancer [94]. At the same line, elevated neutrophil count was found to be correlated with increased mortality in patients with acute myocardial infarction (AMI) [95] and stable coronary artery disease (CAD) [96]. In mouse studies, atherosclerotic plaques of *ApoE*^−/−^ mice showed an increase in CD45^+^ leukocyte content [97]. More specifically, more TC and less BC were found in ApoE^−/−^ mice, indicating the functions of these immune cells [98].

**Table 3:** Representative immune cell subset changes in human diseases

Cells	Changes	Tissues	PMID#
Atherosclerosis (carotid/coronary atherosclerosis, stenosis, cardiovascular death, nonhemorrhagic stroke)			
TC	TC↑, PD-1^+^Tim-3^+^CD8^+^ TC↑, Th17/Th1 TC↑Th1 TC↑, CD4^+^LAP^+^ or CD4^+^CD25^+^ Treg TC↓CD3^+^CD4^+^CD45RA^−^CD45RO^+^CCR7^−^ T(EM) TC↑	PBMC	260352072152475023130116
BC	TC/BC↑	Fibro-fatty (aorta, coronary)	24122585
MC	CD14^++^CD16^+^ MC↑, CD14^++^CD16^−^CCR2^+^ MC↑CD14^+^CD16^++^CCR2- MC↑	PBMC	2299972825012963
MØ	CD86^+^ M1 MØ↑, M2 MØ↑	PBMC, rupture-prone shoulder regions, Adventitial tissue	23078881
Acute myocardial infarction			
TC	CD3^+^CD4^+^CD45RA^−^CD45RO^+^CCR7^−^ T(EM) TC↑HLA-DR^+^ T(EM) TC↑	PBMC	23121518
MC	CD14^++^CD16-CCR2^+^TLR4^+^ MC↑CD14^+^CD16^++^CCR2^+^TLR4^+^ MC↑	PBMC	23121518
Hypercholesterolemia (total serum C levels > 200 mg/dl or 6.5 mmol/L, LDL-C > 160 mg/dl, serum TG ≤ 300 mg/dl)			
TC	CD3^+^ TC↑, CD4^+^ TC↑, CD8^+^ TC↑	PBMC	8546748
MC	CD14^dim^CD16^+^ non-classical MC↑CD64^−^CD16^+^ non-classical MC↑	PBMC	103812988977447
Infection (HCMV with CAP, HIV on stable ART with CAC)			
TC	Lymphocyte↑, CD3^+^ TC↑, CD4^+^ TC↑, CD8^+^ TC↑, Th1 TC↑, CD4^+^CD25^+^Foxp3^+^ Treg TC↓CD8^+^IL-6Rα^low^ T(EM) TC↑, CD8^+^CD57^+^ TC↑	Subintimal PBMC	239689792636053027062409
MC	CD14^++^CD16^+^ MC↑, CD14^++^CD16- MC↑CD14^+^CD16^++^ MC↓, CD14^+^CD16^++^ MC↑	PBMC	2636053024118494
Chronic kidney diseases			
MC	CD14^+^CD16^+^ non-classical MC↑	PBMC	26,877,933
Early rheumatoid arthritis, systemic lupus erythematosus			
TC	CD8^+^CD31^+^CXCR4^+^ TC↑	PBMC	1780453027065298
Obese, pre-diabetes, diabetes mellitus type 2			
TC	NKT-like TC↑, CD3^+^CD56^+^ NKT TC↑	PBMC	24554505
MC	CD14^++^CD16^+^ MC↑, CD14^+^CD16^++^ MC↑	PBMC	21799175
Endarterectomy (CEA and endarterectomy at the femoropopliteal level)			
TC	NKTs↑, TC↑	PBMC, plaques	27051078
DC	CD11b^+^ cDC↑	PBMC, plaques	27051078
MØ	MMP-12^+^CD68^+^ MØ↑	Culprit sections	23316311
Colorectal cancer			
TC	NKT cells↑	PBMC	22220404

### TC subsets

Antigen-specific CD8^+^ TC shows impressive anti-tumor effects [99]. Similarly, there is increasing evidence that both CD4^+^ [93] and CD8^+^ [6, 93, 100] TC are involved in atherosclerosis. More specifically, Th1 and Th17 cell numbers and their related gene expression (T-bet, IFN-γ, STAT4, RORγt, STAT3, and IL-17) were significantly increased in acute coronary syndrome (ACS) patients, whereas the Treg cell population, Foxp3 levels, and plasma IL-10 and TGF-β1 were decreased in ACS patients [6]. NKT-like and NKT cells are a small but significant population of T lymphocytes, and they play an essential role at the very early stages of atherosclerotic plaque development [101]. In atherosclerosis-inducing hyperglycemic diseases (diabetes type 2 or pre-diabetes) [101], endarterectomy at the carotid [91] and colorectal cancer patients [94], the counts of CD3^+^ 56^+^ NKT-like and NKT cells were significantly higher than those of healthy controls. Moreover, there was an increase of the production of granzyme and perforin in these patients.

In atherosclerotic mice, elevated levels of CD4^+^ TC were found in lesions [97, 98]. CD8^+^ TC expressing proinflammatory cytokines (IFN-γ, TNF-α, and IL-12) were also found in the atherosclerotic lesions and spleens of high-fat diet-fed *Ldlr*^−/−^[102] or *ApoE*^−/−^ mice [97]. Importantly, antibody-mediated CD8^+^ TC depletion significantly decreased atherosclerotic plaque formation [102]. More specifically, Th1 were significantly increased in *Ldlr*^−/−^ mice, correlating with increased lesion formation and smooth muscle cell content [103]. Moreover, the amount of Th2 was reduced in atherosclerotic *Ldlr*^−/−^ mice [103]. Furthermore, Th17 quantities were increased in the lesions from *ApoE*^−/−^ mice [104] and in the spleens from obesity mice [105]. In addition, *ApoE*−/− mice exhibit significantly lower numbers of splenic Treg than their wild-type counterparts [106]. Additionally, there was a strong positive correlation between CD4^+^ NKT numbers and markers of inflammation in the plaque (including CD3, T-bet, CCR5, and CCR7), indicating that they promote atherogenesis [91]. Finally, effector memory T cells, including CD4^+^ Th1 and CD8^+^ TC, were associated with increased atherosclerosis and CAD [6].

### BC subsets

Consistent with TC subset changes, TC/BC ratios were found to be positively correlated with fibro-fatty percentage in the plaque [107]. While B2 BC aggravated atherosclerosis, B1a and B1b BC were atheroprotective by secreting natural IgM that increased IgM deposits and reduced necrotic cores in atherosclerotic lesions [108].

Follicular DC (stromal cells) are an important cell type that help with BC maturation. They present antigen in activated germinal centers of primary (BM and thymus), secondary (lymph node and spleen), and artery tertiary lymphoid organs, and contribute to innate and adaptive immune responses in atherosclerosis [109].

### NKC

The CD56^low^CD16^high^ cells comprise the majority of all NKC and are potent mediators of cytotoxicity. By contrast, the CD56^high^CD16^high/low^ cells have low or no cytotoxicity but produce large amounts of various immunoregulating cytokines. Thus, it is not surprising to observe a significant reduction of CD56^low^CD16^high^ NKC and a concomitant loss of NKC function in patients with CAD [110].

### DC

CD11b^+^ cDC, CD11c^+^MHCII^+^CD103^−^CD11b^+^F4/80^−^ cDC, CD11c^+^MHCII^+^CD103^+^CD11b^−^F4/80^−^ cDC, CD11c^+^MHCII^+^CD103^−^CD11b^+^F4/80^+^ mDC, and CD11c^+^CD8^−^CD4^−^ cDC were all found to be expanded in atherosclerotic plaque [91, 111]. In addition, the number of pDC decreased [97].

### MC

MC are the most abundant immune cell type found in atherosclerotic plaques, indicating that they are crucial promoters of atherogenesis. Classical CD14^++^CD16^+^ MC independently predicted cardiovascular events in coronary atherosclerotic subjects [100, 112, 113]. Based on flow cytometry results, the intermediate monocyte subset (CD14^++^CD16^+^) was increased in atherosclerotic patients when compared with that of healthy subjects [93, 100, 112, 114, 115]. Contradictory results were found for non-classical MC subset (CD14^+^CD16^++^): it was observed that they were reduced in patients with CAD, compared with those in healthy donor group [113]; however, a majority of studies showed that they were increased in the patients with coronary plaque vulnerability [115], CKD [116], obesity [114], and hypercholesterolemia [117]. Furthermore, CD40^+^ MC number was found to be a useful biomarker for CKD severity [39] and they were induced in metabolic diseases [17].

We and the others have reported that in mice, CD11b^+^Ly6G^−^Ly6C^middle + high^ MC were consistently and dramatically increased in hypercholesterolemic ApoE^−/−^ mice that were fed with a high-fat diet [118], in *Ldlr*^−/−^ mice [119], in HHcy mice [120], in *miR155*^−/−^ mice [121], and in type 1 diabetes mellitus (T1DM) mice [122]. In addition, resident CD11b^+^Ly6G^−^Ly6C^low^ MC subset in the circulation was reduced in atherosclerotic mice [121].

### MØ

Specific macrophage subsets have been implicated in atherosclerosis, with M1 MØ dominating the rupture-prone shoulder regions of the plaque and M2 MØ dominating the vascular adventitial tissue [123]. In mouse studies, enhanced M1 MØ polarization was observed in left ventricular remodeling after myocardial infarction (MI) [124] as well as in immune organs from hyperglycemic and HHcy mice [122].

### Granulocytes

Over the past couple of years, studies have provided convincing evidence for the presence of neutrophils in atherosclerotic plaques. It was further revealed that neutrophils aggravate endothelial dysfunction [125], recruit MC [126], activate MØ [127], and destabilize plaque [128], which may be attributed to neutrophil-derived reactive species and proteases. Eosinophils may also have a significant role in coronary atherosclerosis since consistent studies have shown a positive association between eosinophil count and increased risk for future cardiovascular events [129]. Eosinophils may exert their pro-atherosclerotic actions through proteins stored in prominent cytoplasmic granules, which may modulate the acute phase response and innate inflammatory response [130]. Similarly, basophils also promote atherosclerosis via these actions [131]. Mast cells are potent immune cells known for their functions in host defense responses and they drive the development of diseases such as asthma and allergies. Mast cells can exert its effects on atherosclerotic plaque progression and destabilization through their release of mast cell-specific proteases chymase and tryptase, growth factors, histamine, and chemokines [132].

## Representative signaling pathways of immune cell subset differentiation

### TC subset differentiation signaling

**Fig. 4:**
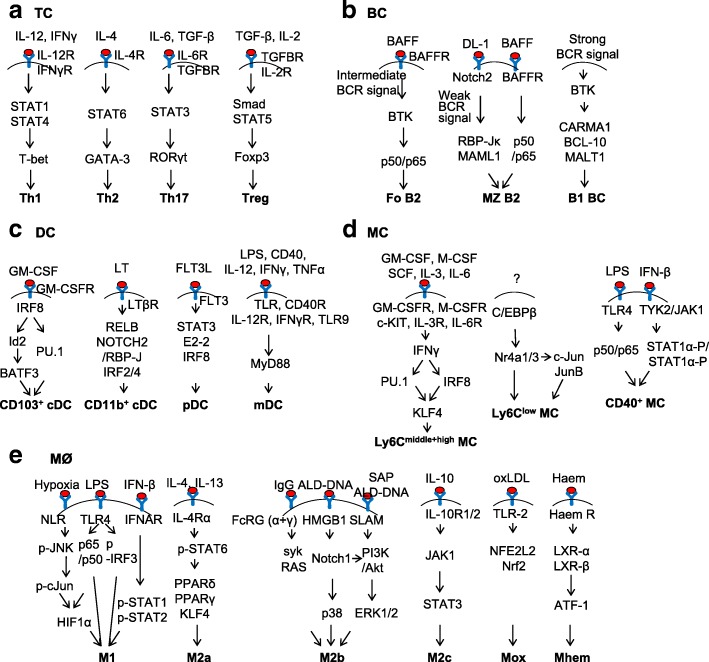
Representative signaling pathways of immune cell subset differentiation. **a** TC. **b** BC. **c** DC. **d** MC. **e** MØ

It is now well established that CD4^+^ TC differentiate into four subsets, which are characterized by their distinct cytokine secretion patterns (Fig. 4a). Among the factors controlling such differentiation are the cytokines present in the milieu of the TC during initial priming. In the case of murine Th1 differentiation, APC-derived IL-12 plays a key role in this respect, as shown by the fact that either IL-12 p40 or IL-12R chain knockout mice had highly impaired Th1 responses [133]. In addition, downregulation of the IL-12Rβ2 chain and thus cessation of IL-12 signaling resulted in alternative differentiation into Th2 cells [134]. IL-12/IL-12R activates STAT4, presumably giving rise to IFN-γ [135]. IFN-γ then activates STAT1/STAT4 and T-bet, which are essential transcription factors for Th1 cell development [136].

IL-4 is the hallmark cytokine that directs Th2 development, since mice deficient in IL-4 or IL-4R failed to develop a potent population of effector Th2 cells [137]. Ligation of IL-4R by IL-4 results in activation of STAT6, which upregulates expression of the Th2 specific transcription factor GATA-3 [138].

The cytokines that are responsible for the differentiation of naïve mouse TC into Th17 are IL-6 and TGF-β [139]. The combination of IL-6 and TGFβ preferentially activates Stat3, which upregulates RORγt, a critical transcription factor for Th17 cells [140]. Multiple functional RORγt binding sites are present in the *Il17A* promoter. Using chromatin immunoprecipitation, RORγt was also found to bind the *Il17A* gene [141].

TGFβ plays an important role in Treg differentiation [142]. It induces phosphorylation of Smad3, which stimulates *Foxp3* transcription by binding to the transcription control elements of *Foxp3* [143]. Treg differentiation is also mediated by IL-2/IL-2R, as IL-2 signaling pathway has been associated with accumulation of Treg in vivo [144]. Upon IL-2/IL-2R activation, phosphorylation of the transcription factor STAT5 appears to play a key role in the generation and expansion of Treg.

### BC subset differentiation signaling

For the transition from immature BC to Fo BC, intermediate level of BCR signal is required (Fig. 4b) [145]. After BCR ligation by antigen, TEC-family protein tyrosine kinase (PTK) BTK5 is recruited and activated [146]. Nuclear factor-κB (NF-κB) is an important downstream effector of BCR/BTK5 signaling [145]. The NF-κB transcription-factor family consists of heterodimers or homodimers of the subunits p50 (NF-κB1), p52 (NF-κB2), c-REL, p65 (RELA), and RELB. The p50/p65 pair determines Fo BC fate. BAFF (B cell-activating factor of the tumor-necrosis-factor family) is also required for Fo BC differentiation. Overexpression of BAFF in transgenic mice induces the production of Fo BC. BAFF engagement activates BTK, which then facilitates BCR-induced activation of the canonical NF-κB pathway.

During MZ BC differentiation, Notch2 interacts with its ligand, Delta-like 1 (DL1), which is specifically expressed by the endothelial cells of red pulp venules in mice [147]. This interaction initiates the cleavage of Notch2, which is not inhibited by weak BCR signaling. The intracellular domain of Notch2 enters the nucleus where it interacts with Mastermind-like 1 (MAML1) and RBP-Jκ transcription factors. This transcriptional complex induces the commitment of BC towards MZ BC [147]. BAFF/BAFF-R interaction also delivers survival signals through canonical NF-KB activation in MZ BCs [148].

A stronger BCR/BTK signal is required for the generation of B1 BC [145], which is supported by the phenotypes of *Btk*-deficient mice [149]. BTK signals through CARMA1, BCL-10 (B cell lymphoma 10), and MALT1 (mucosa-associated lymphoid tissue lymphoma translocation protein 1), which are critical in the development of B1 cells [150]. Interestingly, B1 cell formation is unaffected by impaired BAFF signaling, raising the possibility that elevated BCR signaling in these cells or other microenvironmental factors in the pleural cavity where B1 BC reside may be important for BAFF-independent survival [151].

### DC subset differentiation signaling

Development of CD103^+^ cDC is orchestrated by the transcription factors including inhibitor of DNA binding 2 (ID2), interferon regulatory factor 8 (IRF8), basic leucine zipper ATF-like 3 transcription factor (BATF3), BCL2, and PU.1 (Fig. 4c) [152, 153]. Deletion of any of these genes leads to a severe developmental defect of CD103^+^ DC [70, 154, 155]. The hierarchy and sequential involvement of these specific transcription factors is emerging [153]: IRF8 is required for CD103^+^ cDC differentiation [156]. It was shown that *Irf8*-deficient DC progenitors had reduced expression of several important transcription factors, including ID2 and BCL2 [157]. Id2 is induced in vitro by granulocyte-macrophage colony-stimulating factor (GM-CSF) and is required in vivo for the development of CD103^+^ DC [155]. IRF8 is obligatory for the development of ID2-expressing DC precursors, while BATF3 is induced at later maturation stages of CD103^+^ cDC [70]. It is not clear if all of IRF8’s actions in regulating CD103^+^ cDC development are mediated by BATF3. More likely, IRF8 acts to control both PU.1-dependent and BATF3-dependent target genes in cDC development [153]. Notably, CD103^+^ DC are required for the recruitment of tumor-infiltrating lymphocytes (TILs). It has been shown that melanoma-intrinsic WNT/β-catenin signaling pathway was responsible for inhibiting the crosstalk between TILs and CD103^+^ DC [158]. It was later found that in a tumor model resembling non-T cell-inflamed human tumors, adoptive transferred T cells failed to traffic into tumor site due to the absence of CD103^+^ DC secreting CXCL9 and CXCL10. As a result, TILs highly expressing CXCR3, which is the receptor for CXCL9 and CXCL10, could not be recruited to the tumor site [159]. Taken together, these results indicated that absence of CD103^+^ DC from the tumor microenvironment may be a dominant mechanism of resistance to cancer immunotherapies.

The transcription factors that control general CD11b^+^ cDC development include RELB [160], NOTCH2 [161], RBP-J [162], IRF2 [163], and IRF4 [164]. Of note, IRF4 also controls functional aspects of CD11b^+^ DC, such as their MHC presentation [165] and migration [166]. Consistent with the notion that CD11b^+^ cDC are heterogeneous, deficiency of IRF4 or NOTCH2 only partially impaired CD11b^+^ DC development [161, 166]. In contrast to CD103^+^ cDC, the hierarchy of transcription factors required for CD11b^+^ cDC development is less known [152]. Splenic CD11b^+^ DC were recognized to comprise two subpopulations that are distinguished by ESAM expression [153]. Only the ESAM^+^ subsets of CD11b^+^ cDC are dependent on signaling from the lymphotoxin (LT) β receptor/Notch2/RBP-J, as deletion of *Rbp-J*, using a CD11c-Cre deleter strain in mice led to a 50% reduction in the CD11b^+^ subset of splenic DC [153].

FLT3L and FLT3 constitute the best-characterized growth factor–receptor axis for pDC [167]. STAT3, a key component of the Flt3 signaling pathway, plays a nonredundant role in pDC development [168]. Mice lacking STAT3 have profound reductions in pDC that cannot be rescued by Flt3L administration. In addition, transcription factor IRF8 plays a critical role in pDC differentiation since *Irf8*^−/−^ animals lack pDC [156]. Furthermore, E2-2 directly controls the expression of pDC signature genes, while antagonizing several cDC genes, including *Id2* [169].

The cytokines and factors that control the differentiation of MCs into mDC are less well defined, but key requirements appear to be the recognition of bacterial products through TLR and MyD88 [170] or TC activation signals [171].

### MC subset differentiation signaling

IFN-I signaling in hematopoietic cells is required for the generation of Ly6C^+^ MC (Fig. 4d) [172]. A critical transcription factor that plays a key role in Ly6C^+^ MC differentiation is PU.1. PU.1 is critical for early steps of both myeloid and lymphoid development because PU.1-deficient mice lack MC, granulocytes, and BC [173]. Overexpression of PU.1 leads to activation of IRF8 and Kruppel-like factor 4 (KLF4), which are also key transcription factors involved in Ly6C^+^ MC development [174, 175]. KLF4 is directly regulated by IRF8. Chromatin immunoprecipitation sequencing analysis of the *Klf4* gene locus showed multiple IRF8 peaks [176]. Moreover, it was found that *Irf8*^−/−^ MC progenitors did not express *Klf4* mRNA, and rescue of *Klf4* in *Irf8*^−/−^ mouse myeloid progenitor cells restored MC differentiation. In addition, KLF4 deficiency abolished CCR2 on Ly6C^high^ MC that is a homing chemokine receptor associated with Ly6C^high^ MC migration to tissues upon the sensing of inflammatory stimuli [175].

Transcription factor nuclear receptor subfamily 4, group A, member 1 (NR4A1) is required for Ly6C^−^ MC development [177, 178]. NR4A1 was highly expressed in Ly6C^−^ MC, and Ly6C^−^ nonclassical MC were missing in the blood, BM, and other tissues of *Nr4a1*^−/−^ mice [177, 178]. NR4A1 (also called TR3 or Nur77) is a member of the NR4A family of nuclear receptors, which is considered to function in an anti-inflammatory manner in the vasculature. It was also found that MC from *Nr4a1*^−/−^*Nr4a3*^−/−^ mice contained lower levels of *c-Jun* and *JunB* compared with wild-type mice, indicating that the NR4A family likely regulates expression of *c-Jun* and *JunB*, which are both proto oncogenes, in MC development [174]. The few non-inflammatory Ly6C^−^ MC remaining in the BM of *Nr4a1*^−/−^ mice are arrested in S phase of the cell cycle and undergo apoptosis, implying that NR4A1 functions as a master regulator to control the differentiation and survival of anti-inflammatory Ly6C^−^ monocytes [178].

LPS is a potent inducer of CD40^+^ MC differentiation. The induction of CD40 expression by LPS occurs at the transcriptional level and involves activation of the transcription factors NF-κB and STAT1α. More specifically, LPS directly activates NF-κB and induces endogenous production of IFN-β, which then leads to STAT1α activation, and ultimately CD40 gene expression [179].

### MØ subset differentiation signaling

In respond to various environmental cues, MØ can acquire distinct functional phenotypes via different phenotypic polarization programs (Fig. 4e). M1 differentiation signaling results in the proteosomal degradation of I-κB and the release of NF-κB p65/p50 heterodimer from the NF-κB/I-κB complex [180]. The NF-κB p65/p50 heterodimer is then translocated to the nucleus and binds to the promoters of inflammatory genes. LPS/TLR4 also phosphorylates and activates the transcription factor interferon-responsive factor 3 (IRF3), which are involved in M1 polarization and M1-associated gene induction [181]. These M1-associated genes include type I interferon, such as IFN-β. Secreted type I interferons bind to the type I interferon receptor (IFNAR), which leads to phosphorylation and activation of the transcription factor STAT1 and STAT2. The induced STAT1 and STAT2 then mediate the gene expression of CXCL9 and CXCL10 chemokines [182], which are characteristic of classical M1 MØ activation. In addition to binding to TLRs, some pathogen-associated molecular patterns (PAMPs) and danger-associated molecular patterns (DAMPs), such as hypoxia, are also recognized by a family of cytosolic nucleotide-binding receptors and NOD-like receptors (NLRs) [183]. Upon ligand recognition, NLRs undergo conformational changes and self-oligomerization, which is followed by phosphorylation and activation of JNK and c-Jun MAPKs, both of which are essential for activating hypoxia-inducible factor (HIF) 1α [184]. The crucial role of hypoxia in regulating MØ inflammatory response has been confirmed in mice with myeloid cell-specific deletion of HIF-1α [185], in which HIF-1α was found to be essential in regulating myeloid cell glycolytic capacity, survival and function in the inflammatory microenvironment, which are usually avascular and hypoxic [180]. This is in line with the finding that HIF-1α was induced by NF-κB p65/p50 [186] and plays an important role in modulating MØ phagocytosis of bacteria under sepsis conditions [187].

M2a MØ can be driven by canonical M2a stimuli, such as IL-4 and IL-13 [188]. IL-4 and IL-13 polarize MØs to M2a phenotype via phosphorylation and activation of STAT6 through the IL-4 receptor alpha (IL-4Rα) [180]. The M2a MØ phenotype is then promoted by several transcription factors, including peroxisome proliferator activated receptor γ (PPARγ) [189], PPARδ, and KLF4 [190]. Myeloid-specific deficiency of either PPARγ or KLF-4 resulted in decreased M2a polarization of MØ, leading to accelerated lesion formation in ApoE^−/−^ [191] or Ldlr^−/−^ mice [192]. Moreover, ligation of PPARγ by specific PPARγ ligands preferentially resulted in M2a polarization in mice and in humans [189]. Furthermore, IL-4 and IL-13 strongly increase the production of several different endogenous PPAR ligands, which include 13-HODE, 15-HETE, 15d-GPalpha, and PPAR coactivators (PGC-1), thereby stimulating the PPAR trans-activating activity. Indeed, many of the hallmark IL-4/IL-13-inducible M2 marker genes, such as macrophage mannose receptor (MMR), Arginase I, CD36, dectin-1, depend on PPAR expression [193].

Activated lymphocyte-derived (ALD)-DNA confer MØ M2b polarization via Notch-1/p38 signaling activation [194]. Notch-1 signaling also facilitates ALD-DNA–induced M2b differentiation via PI3K and ERK1/2 pathway [195]. In addition, the M2c MØ phenotype arises in the presence of anti-inflammatory cytokine IL-10 [196]. IL-10 binds to the IL-10R, which is composed of two chains: IL-10R1 and IL-10R2 [197]. IL-10 binding to the receptor activates the tyrosine kinase JAK1, which leads to the phosphorylation of transcription factor STAT3. STAT3 then dimerizes, enters the nucleus, and activates the transcription of anti-inflammatory genes [198]. In murine atherosclerotic plaques, Kadl et al. described a phenotypically distinct MØ subset called Mox [87]. This subset is induced by oxidized phospholipids, such as oxLDL, and protects the organisms from oxidative stress through nuclear factor (erythroid-derived 2)-like 2 (NFE2L2)-mediated expression of antioxidant enzymes such as HMOX1, thioredoxin reductase 1 (Txnrd1), and sulfiredoxin-1 (Sxrn-1). Finally, in human and mouse, haem induces Mhem MØs, which are characterized by increased production of LXRα, LXRβ, haem-induced cyclic AMP-dependent transcription factor 1 (ATF1), HMOX1, and ABCA1 [199].

All the immune cell subsets may interact and communicate with each other. For example, when TGF-β signaling is deficient in CD11c^+^ mDC, there would be increased CD4^+^, CD8^+^, Th1, and Th17 activation and maturation as a result [97]. CD8^+^ TC could also increase monopoiesis and circulating MC levels [102], while Th1 could reduce E06 antibodies that are produced by B1 BC [103]. Moreover, Th17 differentiation relies on IL-6, which is significantly contributed by MØ [105]. Lastly, DC-mediated TLR4/IL-12/IL-10 signaling could polarize Treg, which control GC Tfh-BC axis [200, 201].

## Molecular and cellular modulation of immune cell subsets and its impact on atherosclerosis

**Fig. 5:**
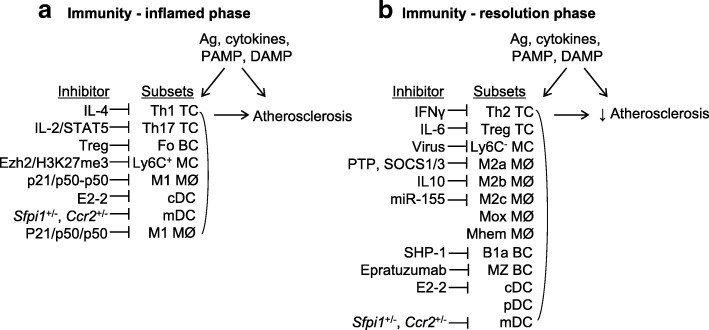
Molecular and cellular modulation of immune cell subsets and its impact on atherosclerosis. Immune cell subsets play different roles in atherosclerosis and can be suppressed by individual endogenous inhibitors as indicated. **a** Inflamed phase in immunity. Inflammatory subsets are induced by antigens (Ag), inflammatory cytokines, pathogen-associated molecular patterns (PAMP), danger-associated molecular patterns (DAMP), in inflamed phase of disease and promote atherosclerosis. **b** Resolution phase in immunity. Anti-inflammatory subsets are induced in resolution phase of disease and suppress atherosclerosis. There are many inhibitors can could interfere with immune cell subset functions

Diverse immune cell subsets in the atherosclerotic plaque strongly contribute to the development of atherosclerotic vascular disease in humans and mice (Fig. 5). During an inflammatory response, increased Th1 TC, Th17 TC, B2 BC, mDC, cDC, Ly6C^+^ MC, and M1 MØ drive atherogenesis. On the other side, atherosclerotic disease risk factors also promote the differentiation of Treg TC, Ly6C^−^ MC, M2a/b/c MØ, Mox MØ, and Mhem MØ, Th2 TC, B1 BC, and pDC, which regulate the inflammatory response and play anti-atherogenic roles during resolution phase [178].

There are many factors that could interfere with immune cell subset functions. Many in vivo and in vitro studies have established a clear role for IL-4 in inhibiting Th1 cell development [202], while Th1-derived cytokine IFN-γ inhibits Th2 proliferation by interfering with Th2 costimulator, IL-1 [203]. IL-2 signaling via STAT5 constrains Th17 cell generation [204], while IL-6 inhibits TGF-β-induced Treg differentiation [205]. Specialized subsets of TC, including follicular Treg and Qa-1-restricted CD8^+^ Treg control Fo BC responses [206]. Humanized LL2 (Epratuzumab), an IgG1 monoclonal antibody, delivers antibody-dependent cellular cytotoxicity to MZ B2 when it binds CD22, which is strongly expressed on MZ BC in lymphoid tissues (“Epratuzumab in Non-Hodgkin’s Lymphomas”, [207]). BC-specific deletion of Shp-1 promotes the development of B1a cells and their expansion in the secondary lymphoid organs [208], indicating that Shp-1 is a key negative regulator of B1a BC activation. Ezh2 was shown to be recruited to the promoters of CCL2 and CCL8 genes in human blood MC, resulting in their gene silencing by H3K27me3, thus controlling the number of inflammatory Ly6C^+^ MC [209]. West Nile virus infection accelerated migrating BM Ly6C^−^ MC differentiation into DC [210]. p21 reprograms M1 MØ by shifting activating p65-p50 to inhibitory p50-p50 NF-κB pathways, and an M2-like status [211]. M2a polarization induced by IL-4 are subject to several negative regulation mechanisms: the SH2-containing tyrosine phosphatases (PTPs) can modulate IL-4 signaling by dephosphorylating JAKs and STAT6 [212], whereas the suppressor of cytokine signaling (SOCS) family, such as SOCS1 and SOCS3, can inhibit the activity of JAKs by blocking the interaction of the JAK catalytic domain with their STAT protein substrates [213]. Importantly, increased IL-10 secretion, accompanied by anti-inflammatory effect exerted by M2a MØ, was found to predominantly impede macrophage M2b polarization [194]. miR-155 represses C/EBP-β/arginase-1 signaling, which is a hallmark of M2c MØ [214]. Transcription factor E2–2 actively maintains the cell fate of mature pDCs and opposes the “default” cDC fate, in part through direct regulation of lineage-specific gene expression programs [169]. Lastly, *Sfpi1*^+/−^ or *Sfpi1*^+/−^ mice had reduced differentiation of GM-CSF-dependent mDCs [215].

## Molecular and cellular modulation of immune cell subsets and its impact on cancer

Tumor-entrained neutrophils (TENs) play a crucial pathophysiological role during cancer development. It has been shown that TENs that are distributed in the pre-metastatic niche in the lung tissues could inhibit metastatic tumor seeding in the lung tissues by generating reactive oxygen species [216]. Mechanistically, tumor-secreted CCL2 was shown to be a critical mediator in this process, which is required for optimal anti-metastatic entrainment of G-CSF-stimulated TENs. In addition, TENs are present in the peripheral blood of breast cancer patients prior to surgical resection, but not in healthy individuals. Importantly, the above mechanism is why CD44 variant-positive ROS-resistant cancer stem-like cells tend to be accumulated in the invasive front of the gastric and breast cancer tissues [217].

Immune-checkpoint inhibitors (ICI) have already emerged as successful therapeutic approaches against multiple cancers [218]. Novel strategies which include promoting immunogenic cell death (such as chemotherapy and radiation) and enhancing antigen presentation by stimulating innate immune responses and dendritic cell function (such as IFN-I and TLR ligands) could potentially promote the formation or the presentation of suitable neo-antigens in tumor tissues that have a non-inflamed, immune cell poor tumor microenvironment. In addition, when combined with ICI, blockage of immunosuppressive factors (such as VEGF, IL-10, and TGF-β), which promotes dendritic cell migration, maturation, and function, might lead to better T cell priming and better immunotherapy against cancers [219].

Myeloid-derived suppressor cells (MDSCs) are a heterogeneous subpopulation of immune cells that are important for inflammatory diseases and cancers [220, 221]. Although MDSCs are present in low quantities in healthy individuals, their numbers are dramatically increased in patients with chronic inflammatory diseases such as cancer, cardiovascular diseases, and autoimmune diseases [220, 222]. MDSCs are generated as a result of sustained and aberrant myeloid cell differentiation. MDSCs are different from terminally differentiated myeloid cells (such as macrophages, dendritic cells, and neutrophils), and they have an activation program that is different from that of mature myeloid cells [223]. MDSCs are characterized by both Gr1 and CD11b markers in mice, which can be used to categorize MDSCs into two subpopulations, which include granulocytic (G)-MDSCs (CD11b^+^Ly6G^+^Ly6C^low^) and monocytic (M)-MDSCs (CD11b^+^Ly6G^−^Ly6C^high^). In humans, G-MDSCs are characterized by CD11b^+^CD14^−^CD15^+^ or CD11b^+^CD14^−^CD66b^+^, whereas M-MDSCs are defined as CD11b^+^CD14^+^HLA-DR^low^CD15^−^ [223]. MDSCs, rather than MC or neutrophils, potently suppress immune responses [220]. It has been shown that depletion of MDSCs results in markedly enhanced anti-tumor immune responses [224, 225]. Thus, MDSCs are promising targets of cancer immunotherapy.

IL-6 is a pleiotropic cytokine with varied systemic functions and it plays a critical role in hematopoiesis as a cofactor in stem cell differentiation and amplification [226]. Clinical studies have shown that upregulated serum IL-6 levels in patients were associated with advanced stages of a number of tumors and short survival in patients [227]. Thus, anti-IL-6 antibodies have been used in the treatment of patients with various cancers and inflammatory diseases. Clinically registered IL-6 inhibitors include anti-IL-6 monoclonal antibody (mAb) (siltuximab) and anti-IL6R mAb (tocilizumab). Blocking IL-6 was proven beneficial towards patients with Castleman disease and inflammatory diseases, and it was well tolerated in cancer patients as well. Nevertheless, IL-6 inhibitors showed no efficacy in large randomized trials of various cancers, in particular plasma cell cancers [228]. One possibility of such failure may be related to our incomplete understanding of the role of IL-6 in regulating the differentiation and function of immune cell subtypes. Future studies are warranted to develop better cytokine-based cancer immunotherapy methods.

## Summary

**Fig. 6:**
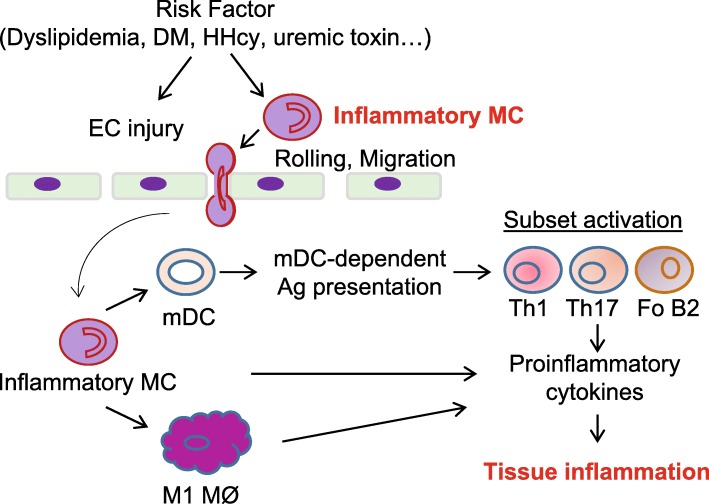
Model of differentiation of inflammatory mononuclear cell, their interaction with endothelial cells and contribution to lymphocyte subset differentiation and tissue inflammation. In early stage of atherosclerosis, Ly6Cmiddle + high monocytes (MC) transmigrate to sub-endothelial space of vessel and further differentiate to M1 macrophages (MØ) and monocyte-derived dendritic cells (mDC). mDC activate T helper cells Th1, Th17, and B2 cells, resulting in chronic inflammation in atherosclerotic plaque. All of these inflammatory mononuclear cell subsets promote atherosclerosis development partially through production of proinflammatory cytokines

Compelling evidence in human and mouse immune cell heterogeneity research points towards a scenario in which immune cell subsets actively change and modulate the development of CVD and cancers. In this review article, we addressed several issues regarding immune cell subset differentiation, which include general hematopoietic cell differentiation processes, subset development, differentiation process and signaling, characterization, disease relevance, and modulation. We elaborated a working model to elucidate the differentiation of pro-inflammatory mononuclear cells, their interaction with endothelial cells, and their contribution to lymphocyte subset differentiation and tissue inflammation (Fig. 6).

## Conclusion

Immune cells can be sub-divided into various subsets that play specialized roles in innate and adaptive immune responses. Immune cell subset differentiation and its complex interaction within the internal biological milieu compose a “pathophysiological network,” an interactive cross-talking complex, which determines and regulates vital conditions of life. Continued research in the understanding of immune cell subsets will provide crucial insights for the etiology, pathobiology, prognosis, and treatment of human CVD and cancers.

## Abbreviations

Ab: Antibody
acLDL: Acetylated low density lipoprotein
ACS: Acute coronary syndrome
Ag: Antigen
AHR: Aryl hydrocarbon receptor
ALD: Activated lymphocyte-derived
Anti-inf: Anti-inflammatory
AP-1: Activator protein 1
APC: Antigen presenting cell
ApoE: Apolipoprotein E
ApoE^−/−^CD11cDNR: Functional inactivation of TGFβ receptor II (TGFβRII) signaling in CD11c + cells
Arg1: Arginase 1
Arg2: Arginase 2
ART: Antiretroviral therapy
ASK1: Apoptosis signal regulating kinase 1
ATF: Activating transcription factor
BAFF: B cell-activating factor of the tumor-necrosis-factor family
Baso: Basophil
BATF3: Basic leucine zipper ATF-like 3 transcription factor
BC: B cell
BCL: B cell lymphoma
BCR: B cell receptor
BDCA2: Blood DC antigen 2
BHA: Butylated hydroxyanisole
BM: Bone marrow
BST2: Bone marrow stromal antigen 2
BTK: Bruton’s tyrosine kinase
C: Cholesterol
C/EBP: CCAAT-enhancer-binding protein
CAC: Coronary artery calcification
CAD: Coronary artery disease
CAP: Carotid atherosclerotic plaque
CCR2: C-C chemokine receptor type 2
CD11cDNR: Functional inactivation of TGFβ receptor II signaling in CD11c + cells
cDC: Classical/conventional dendritic cell
Cdkn2a: Cyclin-dependent kinase inhibitor 2A
CDP: Common dendritic cell progenitor
CEA: Carotid endarterectomy
chol: Cholesterol
CKD: Chronic kidney disease
CLP: Common lymphoid progenitor
CMoP: Common monocyte precursor
CMP: Common myeloid progenitor
CRE: Creatinine
CSF-1: Colony stimulating factor 1
CSFR: Colony-stimulating factor receptor
CVD: Cardiovascular disease
CX3CR1: CX3C chemokine receptor 1
CXCL4: Chemokine (C-X-C motif) ligand 4
DC: Dendritic cell
DC-SIGN: Dendritic cell-specific intercellular adhesion molecule-3-grabbing non-integrin
DIO: Diet-induced obesity
DL-1: Delta-like 1
Dnmt1: DNA methyltransferase 1
EAE: Encephalomyelitis
Eos: Eosinophil
FcγR: Fc receptor-γ receptor
FIZZ1: Found in inflammatory zone 1
Fli-1: Friend leukemia integration 1
Fo: Follicular
FoxP3: Forkhead box P3
Gata-3: GATA binding protein
GC: Germinal center
GM-CSF: Granulocyte macrophage colony-stimulating factor
GMP: Granulocyte-macrophage progenitor
Gran: Granulocyte
haem: Hemoglobin
HCMV: Human cytomegalovirus
HFD: High-fat diet
HHcy: Hyperhomocysteinemia
HIF1α: Hypoxia-inducible factor 1-α
HLA-DR: Human leukocyte antigen – antigen D related
Hlx: H2.0-like homeobox protein
hMDP: Human monocyte-dendritic progenitor
HMGB1: High mobility group box 1
HMOX-1: Heme oxygenase 1
HOX-1: Homeobox-leucine zipper protein
HSV1: Herpes simplex virus 1
IC: Immunocomplex
ICOSL: Inducible T cell costimulatory ligand
ID2: DNA-binding protein inhibitor 2
ID2: Inhibitor of DNA binding 2
IFN: Interferon
IGF: Insulin-like growth factor
IL: Interleukin
IL-1Rα: Interlukin-1 receptor antagonist
Inf: Inflammatory
iNOS: Inducible nitric oxide synthase
IRF: Interferon regulatory factor
KLF: Kruppel-like factor
LCMV: Lymphocytic choriomeningitis virus
Ldlr: Low density lipoprotein receptor
LILRA4: Leukocyte immunoglobulin-like receptor, subfamily A, member 4
LPS: Lipopolysaccharide
LT: Lymphotoxin
LT-HSC: Long-term hematopoietic stem cell
Ly6C: Lymphocyte antigen 6 complex locus C1
lym: Lymphoid
MADS: Metabolite-associated danger signal
MALT1: Mucosa-associated lymphoid tissue lymphoma translocation protein 1
MAML: Mastermind like transcriptional coactivator
MAPK: Mitogen-activated protein kinase
MC: Monocyte
MCMV: Murine cytomegalovirus
mDC: Monocyte-derived DC
MDP: Monocyte-dendritic progenitor
MHC: Major histocompatibility complex
MI: Myocardial infarction
miR: MicroRNA
MM: Multiple myeloma
MMP-7: Matrix metalloproteinase-7
MNC: Mononuclear cell
MØ: Macrophage
MR: Mannose receptor
MZ: Marginal zone
NFAT: Nuclear factor of activated T cells
NFE2L2: Nuclear factor (erythroid-derived 2)-like 2
NF-κB: Nuclear factor κB
NKC: Natural killer cell
NKT: Natural killer T
NØ: Neutrophil
NOI: Nitric oxide intermediates
Nr4a1: Nuclear Receptor Subfamily 4, Group A, Member 1
NSTEACS: Non-ST-elevation acute coronary syndromes
NSTEMI: Non-ST-elevation myocardial infarction
O: Obese
OW: Overweight
oxLDL: Oxidized low density lipoprotein
oxPL: Oxidized phospholipid
PBMC: Peripheral blood mononuclear cell
pDC: Plasmocytoid dendritic cell
PGE2: Prostaglandin E2
PMID: PubMed ID
PPAR: Peroxisome proliferator-activated receptor
PTK: Protein tyrosine kinase
PTP: Phosphatase
RA: Rheumatoid arthritis
RBP-J: Recombination signal binding protein for immunoglobulin kappa J region
Rec: Recombination
ROI: Radical oxygen intermediates
RORγt: Retinoic acid receptor-related orphan receptor γt
SA: Stable angina
SAP: Serum amyloid P component
Siglec: Sialic acid-binding immunoglobulin-like lectin
SIRPα: Signal regulatory protein α
SLE: Systemic lupus erythematosus
SOCS: Suppressor of cytokine signaling
STAT: Signal transducer and activator of transcription
STEAMI: ST-elevation acute myocardial infarction
STEMI: ST segment elevation myocardial infarction
T(EM): Effector memory T cell
T1DM: Type 1 diabetes mellitus
T2DM: Type 2 diabetes mellitus
TC: T cell
TCR: T cell receptor
TD: T cell dependent
Tfh: T follicular helper
TG: Triglyceride
TGFβ: Transforming growth factor β
Th: T helper cell
TH0: Naïve T cell
THBS1: Thrombospondin 1
Throm: Thrombosed
TI: T cell independent
TLR: Toll-like receptor
TNF-α: Tumor necrosis factor-α
Treg: Regulatory T cell
Txnrd1: Thioredoxin reductase 1
UA: Unstable angina
UAP: Unstable angina pectoris
VEGF: Vascular endothelial growth factor
VSV: Vesicular stomatitis virus
α: Anti
